# What if there is no prospective, double blind, randomised trial?

**DOI:** 10.1007/s12471-019-01317-9

**Published:** 2019-08-01

**Authors:** J. R. de Groot

**Affiliations:** Amsterdam University Medical Center, Heart Center, Department of Cardiology, University of Amsterdam, Amsterdam, The Netherlands

Cardiology may be one of the areas in clinical medicine that is best supported by solid evidence, and the majority of recommendations in our national and international guidelines rely on data derived from randomised controlled trials (RCTs). By eliminating bias in treatment allocation, prospective RCTs form the cornerstone of our knowledge base. Nonetheless, RCTs have received their fair share of criticism, for example, that specific groups of patients may have been underrepresented, that the setting is controlled by definition and therefore may not resemble routine clinical care, and that participants in RCTs can be expected to be more motivated and adherent to therapy than may be expected in a routine care setting.

Therefore, to assess the treatment patterns and the real-world uptake of certain therapies or interventions, there is an important role for aggregation of data, and for non-randomised prospective or retrospective investigations. In addition, there may be clinical scenarios that for several reasons may never be studied in a randomised setting. It should be considered that data derived from non-randomised studies are unsuitable to demonstrate a treatment effect, and can merely confirm and never replace the evidence from RCTs, albeit that non-randomised and retrospective studies are extremely valuable to prospectively evaluate standard of care treatment of treatment patterns [1]. Despite the fact that non-randomised research is usually less intrusive for the patients, and certainly less expensive, these types of studies have become increasingly complex recently as a consequence of new European privacy legislation. Subjects enrolled in an RCT or prospective observational study have signed an informed consent regarding the use of their data for clinical research. Retrospective observational research is commonly performed on medical records of standard clinical care data or claim databases, and patients have in general not given specific permission for the use of these clinical data for research. Van der Ree et al. have recently outlined the ridiculous consequences of the implementation of the General Data Protection Regulation (GDPR) for performing a retrospective, non-interventional study on medical records only [2].

This issue of the *Netherlands Heart Journal* features a number of studies in the field of cardiac electrophysiology. What these studies have in common is that all the study settings represent different types of non-randomised, prospective or retrospective analysis.

Tjong and colleagues present a scoping review on temporary pacing in 44,546 patients with mostly atrioventricular block [3]. They conclude that the incidence of complications associated with this commonly applied therapy was 37%, of which 10% were deemed serious. Of note, the rate of complications decreased over time. Almost two thirds of patients were subsequently implanted with a permanent pacing system.

The contribution of Bosman and colleagues describes how the Dutch Arrhythmogenic Cardiomyopathy (ACM) registry is organised, and which type of future research can be expected from that data set [4]. Arrhythmogenic right ventricular cardiomyopathy affects approximately 1:1,000–5,000 people, is characterised by remodelling of the intercalated disk and fibrofatty replacement of myocardium and is the most common form of arrhythmogenic cardiomyopathy (ACM). The rationale for a nation-wide registry is that clinical heterogeneity in the disease and a lack of uniform definitions complicate understanding of the phenotype and thus patient-tailored therapy. Therefore, not only index patients will be included in the Dutch ACM registry, but also first-degree relatives and/or carriers of ACM-associated mutations. As of February 2018, there were 850 individual patient records in the registry. There is no doubt that this initiative will produce valuable insights in the pathophysiology of ACM, even more so because negative controls are available through the inclusion of first-degree relatives known to be mutation-negative, as well as those who refused genetic testing.

Vehmeijer et al. describe the design and rationale of the PREVENTION-ACHD study, investigating the risk of sudden cardiac death (SCD) in adult patients with congenital heart disease (ACHD) [5]. SCD is not so common in these patients, but its prevalence is approximately 25x higher in subjects with ACHD than in age-matched controls. However, risk stratification is immature, and has only be formalised in patients with tetralogy of Fallot. Furthermore, the recommendations for prevention of SCD through the implantation of an ICD are derived from general guideline recommendations in acquired heart disease and poorly predict the occurrence of SCD in ACHD [6]. In PREVENTION-ACHD a novel risk score, consisting of 7 risk factors is introduced and will be tested in a prospective cohort. Patients will be followed up for 2 years for the primary combined endpoint of SCD or sustained ventricular tachycardia/ventricular fibrillation.

Weijs and co-workers present another prospective non-randomised study. These investigators present the 10-year follow-up data of a cohort of 41 patients with atrial fibrillation and no risk factors for stroke, and compare these with 45 control patients in sinus rhythm [7]. The authors show that over the course of follow-up, 63% of patients with atrial fibrillation (AF) died or developed cardiovascular disease, compared with 31% of controls (*p* < 0.001), and that the survival curves diverge further between 5 and 10 years of follow-up. Obviously, age is progressing automatically with prolonged follow-up, and patients aged 56 ± 10 years on average at baseline are expected to become >65 years after 10 years of follow-up. However, the most important driver of increased risk was the development of clinical hypertension. A stunning observation comprises the notion that a large proportion of patients was inadequately dosed with anticoagulation. Only 24 out of 35 AF patients with a CHA_2_DS_2_VASc (congestive heart failure, hypertension, age ≥75 years [doubled], diabetes mellitus, prior stroke [doubled]-vascular disease, age 65–74, sex category) score ≥1 received oral anticoagulation, and the treatment was started within a year from reaching the indication (i.e. reaching an increase CHA_2_DS_2_VASc score) in only 8 of them.

Two papers in this *Netherlands Heart Journal* issue particularly address the use and interpretation of the electrocardiogram (ECG). It has been shown that increased body mass index (BMI) is associated with electrocardiographic changes, but whether BMI should be considered as a continuum in this regard, is not fully understood. Therefore, Hassing and colleagues investigated electrocardiographic data from 1290 volunteers with a normal BMI (18.5–25 kg/m^2^) [8]. They show, with obese subjects as positive controls, that increasing BMI is associated with *P*-wave characteristics on the ECG in particular. Both *P*-wave duration and *P*-wave area increased with increasing BMI, and subsequently PR interval tended to increase too. Interestingly, the QRS axis turned leftward at the same time in an exposure-response relation, and the Sokolow-Lyon voltage criteria decreased. Although of limited absolute magnitude, the ECG changes described underline that patient-specific features, such as BMI, have to be taken into account when interpreting an ECG.

In the retrospective study on interpretation and actions taken following electrocardiography in general practice by Wagenvoort et al., 300 ECGs performed in 14 general practices were adjudicated by an expert panel consisting of an experienced general practitioner (GP) and a cardiologist [9]. There was a high degree of concordance in the judgement of the expert panel with the practitioners who performed the ECGs, but in 1 in 6 ECGs the expert panel had a different interpretation of the ECG. Most commonly, this regarded repolarisation abnormalities, normal ECGs (false-positive GP interpretation) and right ventricular hypertrophy or atrial enlargement. In another 12% there was disagreement between the GPs and the expert panel on the actions taken following the ECG. The authors formulate several learning objectives for the education in ECG interpretation in primary care.

It is obvious that the majority of the studies presented here could or would never have been performed in a prospective, randomised controlled setting. This does not disqualify their results; quite the contrary, the insights provided in these papers will prove their relevance for daily clinical practice.
